# Assignment Through Chiroptical Methods of The Absolute Configuration of Fungal Dihydropyranpyran-4-5-Diones Phytotoxins, Potential Herbicides for Buffelgrass (*Cenchrus ciliaris*) Biocontrol

**DOI:** 10.3390/molecules24173022

**Published:** 2019-08-21

**Authors:** Ernesto Santoro, Giuseppe Mazzeo, Giulia Marsico, Marco Masi, Giovanna Longhi, Stefano Superchi, Antonio Evidente, Sergio Abbate

**Affiliations:** 1 Department Molecular and Translational Medicine, University of Brescia, viale Europa 11, 25123 Brescia, Italy; 2 Department of Sciences, University of Basilicata, Via dell’Ateneo Lucano 10, 85100 Potenza, Italy; 3 Department of Chemical Sciences, Università di Napoli Federico II, Complesso Universitario Monte S. Angelo, Via Cintia 4, 80126 Napoli, Italy

**Keywords:** *Cochliobolus australiensis*, buffelgrass, phytotoxins, absolute configuration, Vibrational Circular Dichroism (VCD), Electronic Circular Dichroism (ECD), Optical Rotatory Dispersion (ORD), DFT (Density Functional Theory) calculations

## Abstract

Radicinin and cochliotoxin (**1** and **2**) two phytotoxic pyranpyran-4,5-diones were isolated together with their close metabolites 3-*epi*-radicinin, radicinol, and its 3-epimer (**3**–**5**), from the culture filtrates of *Cochliobolus australiensis*, a fungus proposed as mycoherbcide for biocontrol of buffelgrass, a very noxious and dangerous weed. The absolute configuration of cochliotoxin was determined by chiroptical Optical Rotatory Dispersion (ORD), Electronic Circular Dichroism (ECD), and Vibrational Circular Dichroism (VCD)) and computational methods. The same methods were used to confirm that of radicinin, radicinol and their 3-epimers, previously determined with chemical, spectroscopic and ECD methods.

## 1. Introduction

In recent years, the use of natural bioherbicides has emerged as one of the most appealing strategies for weed management, due to their low toxicity and, consequently, low impact on the environment and the ecosystem. In particular, fungal phytotoxins have attracted increasing interest, demonstrating to be valuable tools to develop new bioherbicides [1,2,3]. Among them, the pyranpyran-4,5-diones phytotoxins radicinin (**1**) and cochliotoxin (**2**) have recently shown to be promising bioherbicides for the control of buffelgrass (*Pennisetum ciliare* or *Cenchrus ciliaris*) [4], a perennial grass-weed, native of Africa, Southern Mediterranean countries and Middle-Eastern area [5], which has become highly invasive in North America and Australia. This very dangerous weed negatively affects the native vegetation, infesting roadsides and urban landscapes and altering the wildfire regime. Up to-day the only methods used for buffelgrass control are broad-spectrum herbicides and mechanical extirpation. The last one has low efficacy, while glyphosate and imazapyr currently used as herbicides can cause heavy damage to the non-target plants [6] with consequent serious and negative environmental and ecological impact [7]. Therefore, the development of alternative biocompatible methods based on natural compounds like **1** and **2** is particularly relevant.

Radicinin (**1**) was isolated for the first time in 1953 from the fungus *Stemphylium radicinum* [8], but its correct structure was determined only in 1964 by Grove on the basis of chemical derivatizations and spectroscopic analyses [9]. Successively, its production has been reported by a large variety of fungal species, like *Alternaria radicina* [10], *Cochliobolus lunata* [11], *Alternaria chrysantemi* [12], *Alternaria helianthi* [13], *Phoma andina* [14], *Curvularia* sp. [15], *Bipolaris coicis* [16], and *Alternaria petroselini* [17]. Quite often the production of **1** is associated with that of the reduced derivative radicinol (**3**) and of the possible diasteroisomers 3-*epi*-radicinin (**4**), 3-*epi*-radicinol (**5**), and 4-*epi*-radicinol (see Scheme S1 in Supporting Information). More recently, the new dihydropyran-pyran-4,5-dione cochliotoxin (**2**) has been isolated together with **1**, **3** and their 3-epimers **4** and **5** from a liquid culture of the buffelgrass foliar pathogen *Cochliobolus australiensis* [4].

**Figure 1:**
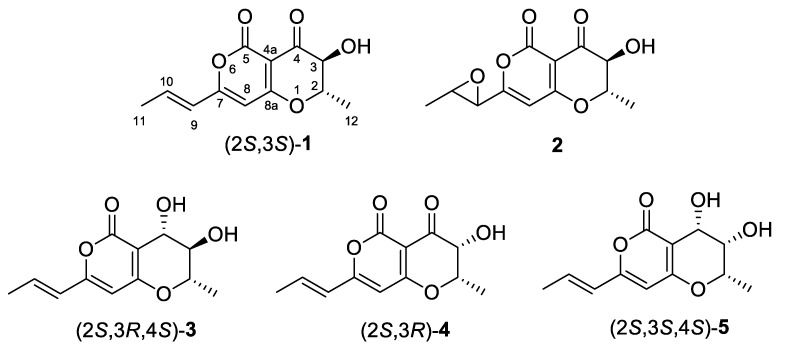
Structure and assigned absolute configuration of radicinin (**1**) and of its structural analogue cochliotoxin (**2**) and of radicinol (**3**), 3-*epi*-radicinin (**4**), and 3-*ep*i-radicinol (**5**). Compounds **1** and **2** are treated in the text, while the chiroptical data of compounds **3**–**5** are treated in the Supplementary Material.

Radicinin (**1**) (Figure 1) demonstrated high target-specific toxicity on buffelgrass, low toxicity to native plants, and no teratogenic, sub-lethal, or lethal effects on zebrafish (*Brachydanio rerio*) embryos [18]. It also showed antifungal, insecticidal, and plant growth regulatory activity [12], as well as antibiotic activity against Gram-positive bacteria, including *Staphylococcus aureus* and *Clostridium* sp. [19]. More recently, the ability of **1** to inhibit *Xylella fastidiosa*, the causal agent of many devastating plant diseases, including Pierce’s Disease of grapevine, phony peach disease, alfalfa dwarf disease, plum leaf scald, citrus variegated chlorosis, and leaf scorch of almond, coffee, elm, oak, oleander, pear, and sycamore was reported [20]. The wide and specific bioactivity of **1** and **2**, which allows one to envisage their promising application as both biopesticides and pharmaceutical compounds, stimulating thus the development of studies on their structure-activity relationship [4,18] and on their large-scale production by fungal fermentation or through their total stereoselective synthesis. A rapid and sensitive HPLC method for quantification of radicinin in complex mixtures has been recently developed and its production by different *C. australiensis* strains and in different cultural conditions has been evaluated [21]. The further studies obviously cannot overlook the influence of the stereochemistry of the compounds, taking into account the well-known close relationships between relative and absolute configuration and biological activity of phytotoxins [22,23].

As far as the stereochemistry of these compounds is concerned, their relative configuration was usually determined by ^1^H-NMR spectroscopy, while the absolute configuration (AC) was determined by chiroptical or diffractometric methods. In particular, (2*S*,3*R*,4*S*) AC was assigned to (−)-radicinol (**3**) (Figure 1) isolated from *Cochliobolus lunata* by applying the exciton chirality method [24] to account for the electronic circular dichroism (ECD) spectrum of its 3,4-*bis*-*p*-chlorobenzoate [11]. This allowed, in the same paper, to assign also the (2*S*,3*S*) configuration to **1**, from which **3** and its (4*R*) epimer 4-*epi*-radicinol (**5**), were obtained by carbonyl reduction with NaBH_4_. However, this assignment appeared questionable, because appearance of other transitions allied to the α-pyrone chromophore in the couplet wavelength region rendered the dibenzoate rule inapplicable. Therefore, the relative and absolute configuration of **1** was later definitively confirmed by X-ray analysis of the natural toxin and its 4-*p*-bromobenzoate, respectively [12]. Reduction of (−)-**1** with NaBH_4_ provided (−)-**3**, thus allowing to confirm also its (2*S*,3*R*,4*S*) AC. The AC of 3-*epi*-radicinin (**4**) and 3-*epi*-radicinol (**5**) isolated from *B. coicis* culture was established by derivatizing **4** to its 3,4-*bis*-*p*-bromobenzoate [16] and applying to the ECD spectrum of latter the exciton chirality rule [24]. Thus AC (2*S*,3*S*,4*S*) was assigned to (−)-**5** and (2*S*,3*R*) to (−)-**4** from which (−)-**5** (Figure 1) is obtained by carbonyl reduction with NaBH_4_ [16]. Also in this case the AC assignment based on application of the exciton chirality rule appeared quite uncertain because, as reported by the authors [16]: Indeed only one of the expected couplet components was visible, due to spectral overlap with transitions allied to the α-pyrone chromophore. The stereo-structural relationship between **1** and **4** was also demonstrated by the results of a biosynthetic study carried out administrating (**–**)-deoxyradicinin to *B. coicis* culture [25]. For cochliotoxin (**2**) only the (2*S*,3*S*) AC at the pyranone moiety was determined by applying the advanced Mosher’s method [26] through esterification of its C-3 hydroxy group [4]. However, only the *trans*-relative configuration to the 9,10-oxyrane ring was assigned by ^1^H-NMR data of **2** [4] while the AC of this moiety was still unknown. Interestingly, *trans*-9,10-epoxydes of both **4** and 4-*epi*-radicinol 4-*O*-methylether have been isolated from fungal cultures of *B. coicis* [16] and *A. chrysantemi* [27] respectively, but also in those cases the AC of the epoxide ring was not established.

We then undertook an investigation aimed at establishing AC of all the stereocenters of natural cochliotoxin (**2**) (Figure 1) by ab initio computational analysis of chiroptical spectra, i.e., optical rotatory dispersion (ORD), electronic circular dichroism (ECD), and vibrational circular dichroism (VCD) [28,29,30,31,32]. We also considered worthwhile to apply the same approach to the closely-related compound **1**, and compounds **3**–**5** (reported in SI) with the aim to confirm the AC reported in the literature and, eventually, to test the reliability of the approach on this class of natural products. In fact, this approach demonstrated to be particularly straightforward and reliable for the AC assignment of natural phytotoxins [33,34,35,36] and plant metabolites [37,38,39], allowing to assign AC faster than the chemical correlation and diffractometric methodologies and more reliably of the exciton chirality approach employed in literature on these pyranpyran-4,5-diones phytotoxins.

## 2. Results and Discussion

### 2.1. Experimental VCD-IR, ECD-UV, and ORD

**Figure 2:**
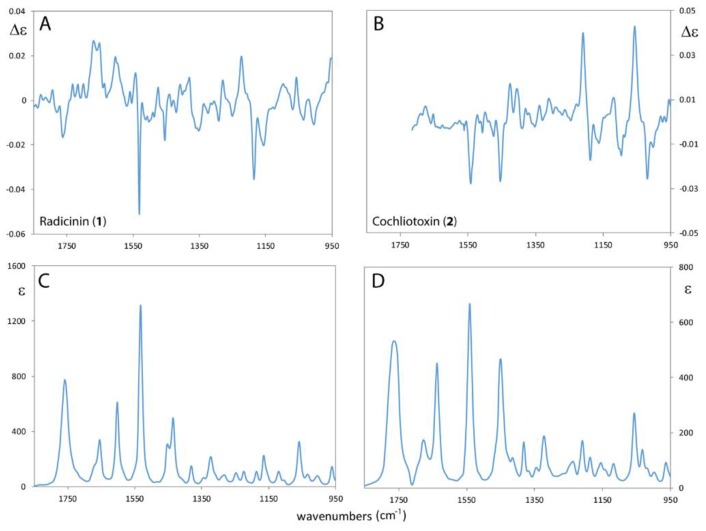
Experimental Vibrational Circular Dichroism (VCD) and IR spectra of radicinin (**1**) (panels **A**,**C**) respectively and of cochliotoxin (**2**) (panels **B**,**D**) in CDCl_3_ solution. (See Figure S1 of Supplementary Material for the VCD noise level).

The VCD and IR spectra of **1** and **2** were measured in the 950–1850 cm^−1^ range as deuterated chloroform (CDCl_3_) solutions (Figure 2).

The IR spectra of **1** and **2** are quite similar, except for the exact frequency position of the bands in the 1650–1600 cm^−1^ range and for the band at 1450 cm^−1^. The former bands (at ca. 1600 cm^−1^ for **1** and 1640 cm^−1^ for **2**) are associated (by computational vibrational analysis, vide infra) to modes involving the C7=C8 stretching mode. Due to the conjugation with exocyclic propylene moiety the band is located at lower frequency in radicinin (**1**) than in the epoxyde derivative **2**. Furthermore in cochliotoxin (**2**) a weak IR band at ca. 1210 cm^−1^ can be also noted, which is not present in radicinin (**1**), associated with CO-symmetric stretching of the oxyrane C-O-C. Both VCD spectra, in the fingerprint region, possess some identifiable similar features: the two negative bands at ca. 1530 cm^−1^ and 1454 cm^−1^, allied to C-O and C4a=C8a stretching modes, and the (−,−,+) group of bands at ca. 1150 cm^−1^, from lower to higher frequency, in which olefinic C-H in-plane bendings are involved.

**Figure 3:**
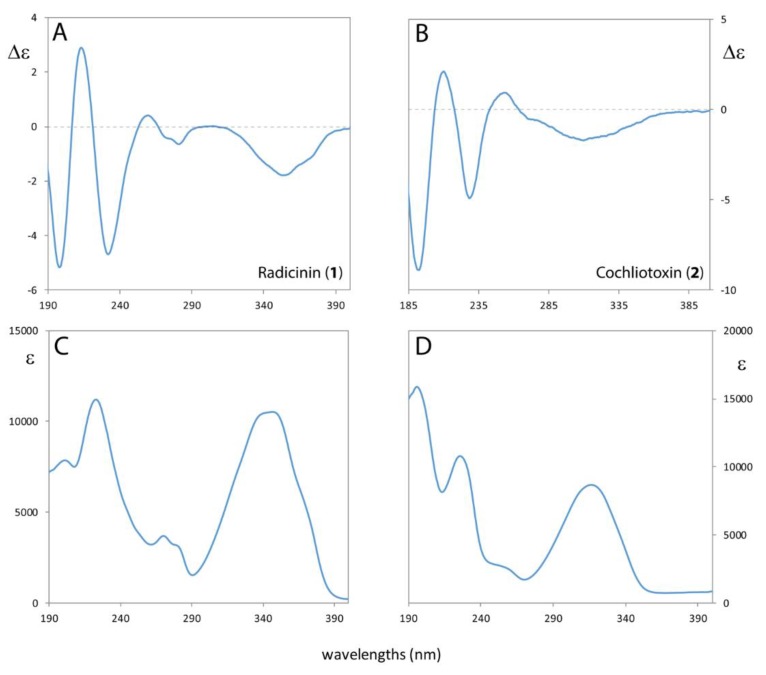
Experimental ECD and UV spectra of radicinin (**1**) (panels **A**,**C**) and of cochliotoxin (**2**) (panels **B**,**D**) in acetonitrile solution.

The UV and ECD spectra of **1** and **2** were measured on acetonitrile solutions in the 190–400 nm range (Figure 3). The UV spectra exhibit two bands centered at about 200 and 220 nm respectively (for **1** the 200 nm band is less intense), a weak band at ca. 270 nm (slightly structured for **1**) and a broad intense band at ca. 340 nm for **1** and at ca. 320 nm for **2**. This low-lying transition is ascribed to enedione transition; [40] the tri-enedione transition of radicinin (**1**) is clearly at higher wavelength with respect to the di-enedione transition of cochliotoxin (**2**) due to the more extended conjugation. The experimental ECD spectra of the two compounds show quite similar profiles: three Cotton effects (CEs) in the 190–250 nm range (negative at 197 nm, positive at 212 nm, and negative at 230 nm), a weak negative band is centered at ca. 270 nm and a broad negative band may be also attributed to the low lying enedione transition.

Experimental ORD of **1** and **2** were measured in chloroform solutions. Both ORD trends are negative in the range of ca. −158 (at 589 nm) and −936 (at 405 nm) for **1** and −131 (at 589 nm) and −594 (at 405 nm) for **2** (see Table 1). It is worth noting that the ORD negative trend is higher in magnitude for radicinin (**1**) with respect to cochliotoxin (**2**) because of the low lying electronic transition of **1** (Figure 3) which is more red shifted than **2** and more contributes (via the Kronig-Kramers transform [41,42]) to the OR values at the four different wavelengths.

### 2.2. Absolute Configuration Assignment

To test the reliability of the computational analysis of chiroptical data for the AC assignment of naturally occurring pyranpyran-4,5-diones phytotoxin cochliotoxin (**2**) we first applied this approach to chiral phytotoxins of known AC radicinin (**1**). As reported in the introduction, the (2*S*,3*S*) AC of (−)-**1** has been unequivocally established by X-ray analysis of its 4-*p*-bromobenzoate [12], thus confirming a previous AC assignment [11] based on exciton chirality approach and by Mosher method [4] respectively, which appeared questionable. Therefore, we carried out a conformational computational analysis for both molecules **1** and **2** assuming (2*S*,3*S*) AC for the common pyranone moiety. About cochliotoxin 9,10-oxyrane ring, only the *trans*-relative configuration was established by ^1^H-NMR data [4] while the AC of this moiety was still unknown. Therefore, the computational analysis was performed on both possible diastereoisomers (2*S*,3*S*,9*R*,10*S*)-**2** and (2*S*,3*S*,9*S*,10*R*)-**2** having the oxyrane moiety in *trans* conformation. The conformational analysis at the Molecular Mechanics (MM) level (see paragraph 3.5 for computational details) provided 6 distinct populated conformers for all three molecules (2*S*,3*S*)-**1**, (2*S*,3*S*,9*R*,10*S*)-**2** and (2*S*,3*S*,9*S*,10*R*)-**2**. To take into account possible solvation effects, all conformations found by MM were fully optimized in acetonitrile (for ECD-UV calculations) and chloroform (for VCD-IR and ORD calculations) using IEFPCM solvation model [43] by density functional theory (DFT) at DFT/B3LYP/TZVP level for VCD-IR, TD-DFT/CAM-B3LYP/aug-cc-pVDZ for ECD-UV and TD-DFT/B3LYP/aug-cc-pVDZ for ORD calculations. The geometry optimizations provided two most populated conformers for radicinin (2*S*,3*S*)-**1** (with one of them at almost 90% of the overall population), two conformers for (2*S*,3*S*,9*R*,10*S*)-**2** and three for (2*S*,3*S*,9*S*,10*R*)-**2**. In the last two cases just one conformer was found, at ca. 99% of the overall population (see Table S1 and Figure S1 in SI for details).

**Figure 4:**
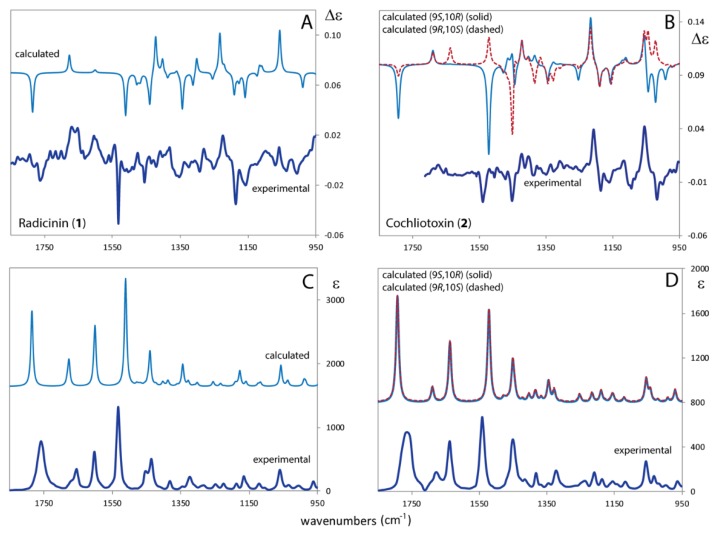
Comparison of experimental and calculated VCD and IR spectra of radicinin (**1**) (panels **A**,**C**) and of cochliotoxin (**2**) (panels **B**,**D**). For (**2**) the two possible diastereomers have been calculated. DFT/B3LYP/TZVP/PCM(CHCl_3_) level of theory. Applied scaling factor is 0.99. Calculated IR spectra are divided by factor of 3.

In Figure 4 we report the comparison of the experimental and calculated VCD-IR spectra (1850–950 cm^−1^ range) of **1** and **2**, the calculation for the former molecule being for the (2*S*,3*S*)-**1** choice, the calculation for the latter being for the two diastereomeric forms (2*S*,3*S*, 9*R*,10*S*)-**2** and (2*S*,3*S*,9*S*,10*R*)-**2**. The prediction of the experimental VCD-IR spectra of radicinin (**1**) (Figure 4, left panels) is qualitatively satisfying. In particular, the two negative bands at ca. 1530 cm^−1^ and 1450 cm^−1^, associated to C=O and C4a=C8a stretching modes, are well predicted in sign and intensity. Also the (−,−,+) triplet at ca. 1150 cm^−1^ (allied to olefin C-H in-plane bendings) is satisfactorily predicted. Let us now turn to the comparison of experimental and calculated VCD-IR spectra of (9*R*,10*S*)-**2** and (9*S*,10*R*)-**2** (Figure 4, right panels). The calculated VCD curve for (9*S*,10*R*)-**2** better qualitatively predicts the experimental VCD spectrum. In fact, the (9*R*,10*S*)-**2** isomer does not correctly predict the experimental negative band at ca. 1540 cm^−1^ and at ca. 1000 cm^−1^; thus we strongly support the (2*S*,3*S*,9*S*,10*R*)-**2** as the AC correct of cochliotoxin (**2**). Discrimination of the two selected AC is given by the VCD negative bands at ca. 1550 cm^−1^ and by the structured (+,−) doublet (from higher to lower frequency) at ca. 1050 cm^−1^, while the (+,−,−) triplet and the negative band followed by two positive features at ca. 1450 cm^−1^ are calculated with equal or at least similar efficacy with the two AC choices. The discriminating power of DFT calculated VCD and IR spectra (the latter type being calculated almost the same with the two AC choices) is critically dependent on the value of the chosen scaling factor, which is forcibly equal on all the investigated spectroscopic region and a bit too high (0.99); further investigation on this aspect is beyond the scope of this work. Recently a paper appeared for conducting the comparison of experimental and calculated spectra taking into account not only experimental errors (reported for VCD in Figure S11) but also the uncertainties of the ab initio calculations, however such methodological estimation is beyond the scope of this work [44].

**Figure 5:**
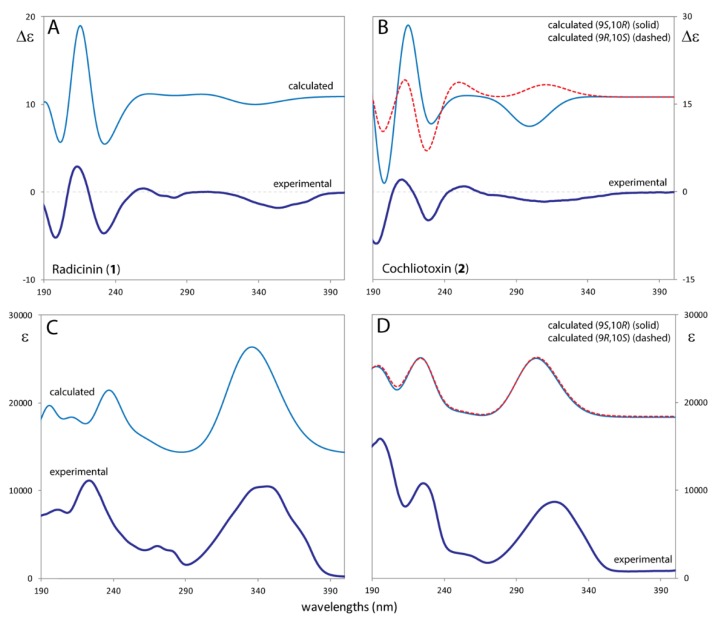
Comparison of experimental and calculated Electronic Circular Dichroism (ECD) and UV spectra of radicinin (**1**) (panels **A**,**C**) and of cochliotoxin (**2**) (panels **B**,**D**). For (**2**) the two possible diastereomers have been calculated. TD-DFT/CAM-B3LYP/aug-cc-pVDZ/PCM(ACN) level of theory. Calculated spectra are red-shifted by 15 nm and divided by factor 3.

The comparison between the experimental and computed UV and ECD spectra is reported in Figure 5. For a better comparison with experimental spectra, the calculated ones were divided by a factor 3. Calculation of radicinin (**1**) ECD-UV spectra provide a good prediction of the experimental ones in signs and relative positions of the bands.

By the side of cochliotoxin (**2**), we compared ECD-UV calculated spectra of (9*R*,10*S*)-**2** and (9*S*,10*R*)-**2** diastereomers. As reported in Figure 5 (right panels), despite the calculated UV spectra of the two diastereomers are practically coincident, the ECD profiles are slightly different especially with regard to the low-lying transition at ca. 320 nm. (9*R*,10*S*)-**2** isomer has a positive calculated CE at ca. 330 nm, while its (9*S*,10*R*)-**2** counterpart has a negative ECD band, consistent with experiment. As reported above, this transition was attributed to the di-enedione-like chromophore which appears to be sensitive to the chirality of carbon 9 and, as a matter of fact, it becomes a highly discriminating electronic transition for the AC assignment of **2**. These considerations about ECD spectra support the previous assignment based on VCD-IR, establishing that the AC of cochliotoxin (**2**) is(2*S*,3*S*,9*S*,10*R*)-**2**.

The AC of **1** and **2** was also investigated by computing OR values at several wavelength and comparing to the corresponding experimental values (we call this set of OR values somewhat inappropriately ORD curve). The experimental negative trend of radicinin (**1**) is well predicted by calculation of optical rotatory power at four different wavelengths (Table 1) confirming the (2*S*,3*S*)-**1** AC. On the other side the (9*S*,10*R*)-**2** diastereomer of cochliotoxin (**2**) has negative ORD trend sign which is consistent with the experimental measured in chloroform solution (though a bit higher than observed), while calculated (9*R*,10*S*)-**2** ORD shows positive trend. It is clear that also ORD calculation supports (2*S*,3*S*,9*S*,10*R*)-**2** as the AC of cochliotoxin.

## 3. Materials and Methods

### 3.1. General Experimental Procedures

^1^H-NMR spectra were recorded at 400 MHz in CDCl_3_ on a Bruker (Karlsruhe, Baden-Württemberg, Germany) spectrometer. The same solvent was used as internal standard. ESIMS (ElectroSpray Ionization Mass Spectroscopy) spectra were recorded on an Agilent 6120 Quadrupole LC/MS instrument (Agilent Technologies, Milan, Italy). Analytical and preparative TLC (Thin Layer Chromatography) were performed on silica gel (Kieselgel 60, F254, 0.25 and 0.5 mm respectively) plates. The spots were visualized by exposure to UV radiation (253 nm) or by spraying first with 10% H_2_SO_4_ in MeOH and then with 5% phosphomolybdic acid in EtOH, followed by heating at 110 °C for 10 min. Column chromatography was performed using silica gel (Merck, Kieselgel 60, 0.06–0.200 mm).

### 3.2. Fungal Strains

The *Cochliobolus australiensis* (LJ-4B) strain used in this study was isolated from the same infected plant collected near La Joya, Hidalgo County in south Texas, USA, in September 2014. The isolate is maintained on potato dextrose agar (PDA, Fluka, Sigma-Aldrich Chemic GmbH, Buchs, St. Gallen, Switzerland) and stored at 4 °C in the strain collection of S. Meyer at the USFS RMRS Shrub Sciences Laboratory, Provo UT, USA.

### 3.3. Isolation of Fungal Metabolites

The fungal metabolites **1**–**5**, have been isolated from in vitro PDB cultures of *C. australiensis* according to procedures previously published and compared by TLC (carried out using different solvent systems) to the standard compounds [4]. Furthermore, their purity was >98%, as checked by ^1^H-NMR and LC-MS.

### 3.4. Chiroptical Spectroscopies

VCD spectra were taken from 950 to 1800 cm^−1^ on a Jasco FVS6000 FTIR spectropolarimeter. Solutions spectra were recorded on ca. 0.05 M solutions in CDCl_3_ solvent in 200 μm BaF_2_ IR cells. Five thousand scans were taken for each measurement. VCD spectra of the solvent were recorded in the same experimental conditions and then subtracted. All data are reported in ∆ε (M^−1^cm^−1^) vs. ν (cm^−1^), from knowledge of the cell pathlength and solution concentration.

ECD spectra were taken from 400 to 185 nm on a Jasco 815SE spectropolarimeter. Solutions at ca. 0.0045 M in CH_3_CN solvent were employed and were contained in 0.1 mm quartz cylindrical cuvettes. For each measurement, 10 scans were taken and averaged. ECD spectra of the solvent in the same experimental conditions were subtracted. Data are reported in ∆ε vs. λ (nm), from knowledge of the cell pathlength and solution concentration.

The ORD measurements were carried out with a Jasco P-2000 Polarimeter. A 10 cm SiO_2_ cuvette was employed in all cases with CHCl_3_ solutions at ca. 0.35 g/100 mL. Solutions were studied at 25 °C and five wavelengths were considered for Optical Rotations (OR), 589 nm (Na lamp), 546, 435, and 405 nm (Hg lamp). OR data were obtained with ten measurements at each wavelength and proper subtraction of the OR data of the solvent at the same wavelength was carried out. Specific rotation values were obtained from a program of the instrument software. The experimental data at two adjacent wavelengths were connected through a straight line.

### 3.5. Computational Details

Conformational analysis of **1** and (9*R*,10*S*)-**2**, (9*S*,10*R*)-**2** was carried out at the Molecular Mechanics (MM) level, with allowance of all conformers in the range of 20 kcal/mol from the most stable one. All these conformers were fed to Gaussian09 [45] and DFT calculated conformers and IR and VCD spectra were obtained at B3LYP/TZVP within the PCM approximation [43]. All chiroptical properties were calculated as Boltzmann averages, weighed with conformer population factors obtained according to free energy ∆G at T = 298 K. VCD-IR spectra simulation was obtained by assigning a Lorentzian band to each calculated transition, with assigned bandwidth of 10 cm^−1^, for the rotational and dipole strengths calculated through Gaussian09. Scaling factor of 0.99 was applied to the calculated wavenumbers of IR and VCD bands. ECD calculated spectra were obtained by Gaussian09 using CAM-B3LYP/TZVP level of theory. ECD Spectra were simulated by assigning to each electronic calculated transition with 0.2 eV wide Gaussian bands. All calculated ECD and UV absorption transitions were shifted by 15 nm. The first 50 excited states were considered in setting up the calculation. OR values were calculated at B3LYP/aug-cc-pVDZ level of theory at 589, 546, 435, and 405 nm.

## 4. Conclusions

The assignment of the AC is a very important structural feature, since AC is closely related to biological activity [22,23]. Several phytotoxins and in particular those of fungi proposed as mycoherbicides and thus with potential practical application, are produced in very low amount. This is a great limiting feature for their suitable formulation and use in agriculture as the case of the pyrapyrandiones reported in this manuscript. Thus a solution is the development of their total enantioselective synthesis, with good yield and economic and eco-compatible process. As an alternative to stereo-controlled synthesis, the assignment of AC can be pursued efficaciously, with employment of a thorough set of chiroptical methods (VCD, ECD, and ORD) and of DFT calculations, as done here. Indeed with this approach not only did we confirm the AC of radicinin (**1**), [11] but we assigned AC de novo for cochliotoxin (**2**), and we confirmed the AC assignment for radicinol (**3**), *epi*-3-radicinin (**4**), and *epi*-3-radicinol (**5**), which had been previously characterized [16].

## Figures and Tables

**Table 1:** Comparison of Experimental and Calculated Specific OR Values at Four Different Wavelengths for Radicinin (**1**) And Cochliotoxin (**2**). Experimental ORD was Measured in Chloroform Solvent. TD-DFT/B3LYP/aug-cc-pVDZ/PCM(CHCl_3_) Level of Theory.

	[α] Radicinin (**1**) (CHCl_3_)		[α] Cochliotoxin (**2**) (CHCl_3_)		
(nm)	Calc.	Exp.	(9*R*,10*S*) calc.	(9*S*,10*R*) calc.	Exp.
589	−206	−159	+128	−366	−131
546	−259	−231	+171	−459	−168
435	−605	−556	+504	−1010	−405
405	−885	−936	+789	−1371	−595

## Supplementary Materials

Supplementary Information (SI) is available online. It reports experimental and calculated ECD, ORD and IR and VCD spectra of radicinol (**3**), 3-*epi*-radicinin (**4**) and 3-*ep*i-radicinol (**5**). Full computational analysis of the three compounds is also provided.
