# Supplementary material for: Assignment Through Chiroptical Methods of The Absolute Configuration of Fungal Dihydropyranpyran-4-5-Diones Phytotoxins, Potential Herbicides for Buffelgrass (Cenchrus ciliaris) Biocontrol
Source: Molecules. 2019 Aug 21;24(17):3022. doi: 10.3390/molecules24173022 (PMC6749493; doi:10.3390/molecules24173022)
Supplement: Supplementary file 1 [file molecules-24-03022-s001.pdf]

Absolute configuration of fungal dihydropyranpyran-4-5-diones phytotoxins: potential herbicides for buffelgrass (*Cenchrus ciliaris*) biocontrol

Ernesto Santoro[1,2,†], Giuseppe Mazzeo[1,†], Giulia Marsico[2,†], Marco Masi[3,†], Giovanna Longhi[1], Stefano Superchi[2,*], Antonio Evidente[3,*] and Sergio Abbate[1,*]

SUPPORTING INFORMATION

With Addition of:

Absolute configuration assignment of radicinol (3), 3-*epi*-radicinin (4), 3-*epi*-radicinol (5) via comparison of experimental and calculated VCD, ECD and ORD spectra.

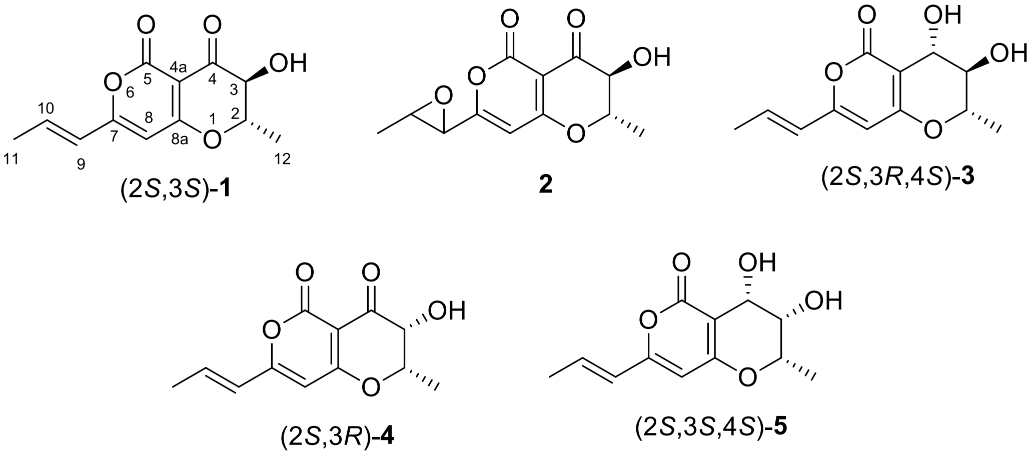

Scheme S1. Structure and assigned absolute configuration of radicinin (**1**), and its structural analogues radicinol (**3**), 3-*epi*-radicinin (**4**), 3-*epi*-radicinol (**5**) and cochliotoxin (**2**). **1** and **2** are discussed in the text, data and calculations for **3**, **4** and **5** are given only in this SI file. (Sections SI-1 through SI-3)

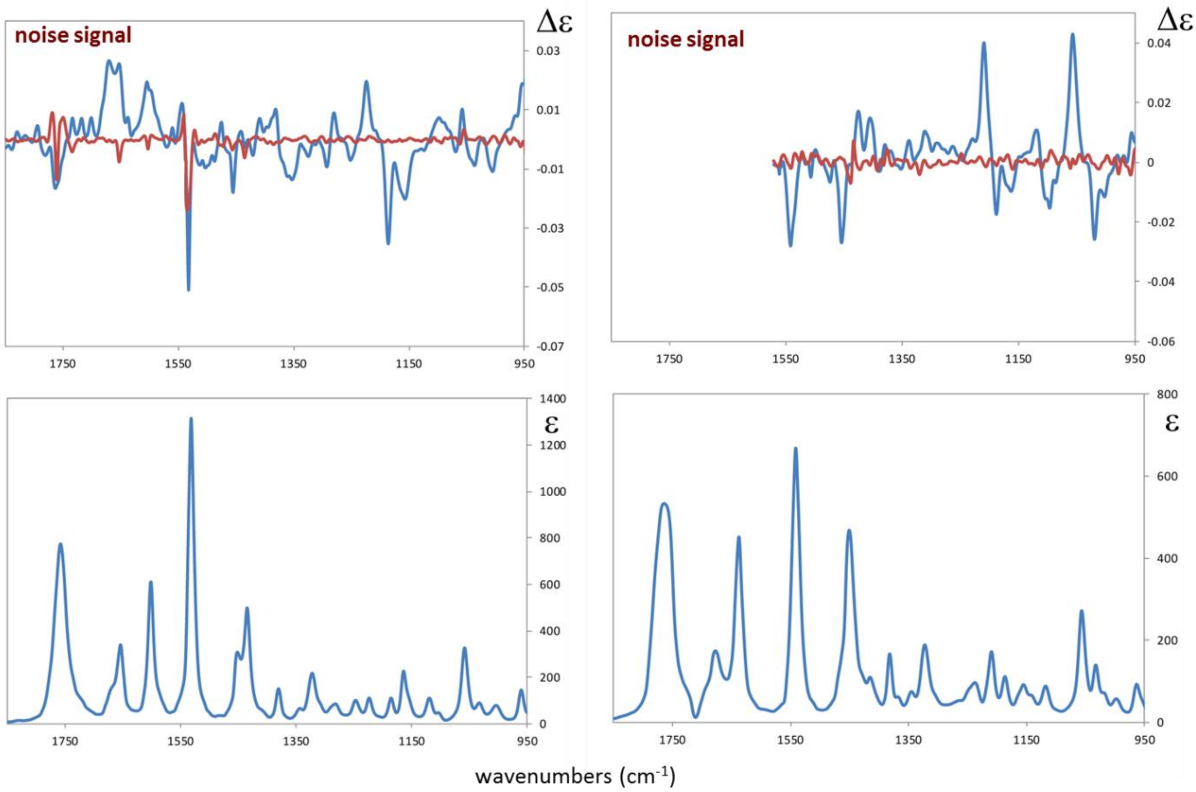

Figure S1. Experimental VCD (upper panels) and IR (lower panels) spectra of radicinin (1) (left) and cochliotoxin (2) (right) in $CDCl_3$ solution. Noise level signal curves are reported in red solid lines.

Table S1. Conformers relative ΔG energies (in kcal/mol units) and percent population factors (computed from the Boltzmann distribution) for radicinin (**1**) and cochliotoxin (**2**). The 3D-structures of the compounds are given in Figure S1.

Radicinin ($CHCl_3$)

**Conformers**	**ΔG**	**% pop**
**1**	0.00	89.2
**2**	1.12	10.8

Radicinin (ACN)

**Conformers**	**ΔG**	**% pop**
**1**	0.00	81.2
**2**	0.87	18.8

(9*S*,10*R*)-cochliotoxin ($CHCl_3$)

**Conformers**	**ΔG**	**% pop**
**1**	0.00	99.9
**2**	3.98	0.1

(9*S*,10*R*)-cochliotoxin (ACN)

**Conformers**	**ΔG**	**% pop**
**1**	0.00	99.7
**2**	3.47	0.3

(9*R*,10*S*)-cochliotoxin ($CHCl_3$)

**Conformers**	**ΔG**	**% pop**
**1**	0.00	99.0
**2**	2.86	0.8
**3**	3.69	0.2

(9*R*,10*S*)-cochliotoxin (ACN)

**Conformers**	**ΔG**	**% pop**
**1**	0.00	98.4
**2**	2.57	1.3
**3**	3.36	0.3

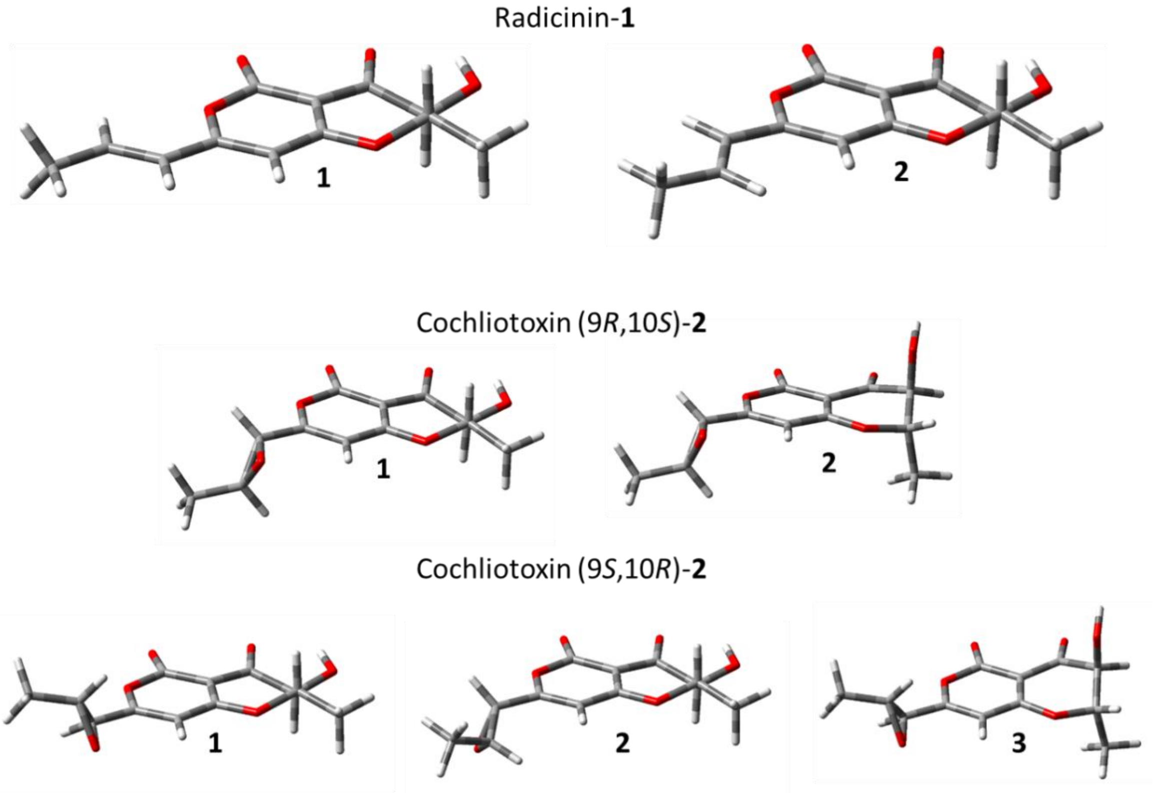

Figure S2. Calculated conformer structures of radicinin-1 and cochliotoxin-2.

Section SI-1. <u>AC of Radicinol (**3**)</u>

Conformational search and DFT optimization at B3LYP/TZVP level of theory (PCM($CHCl_3$) was used for VCD and ORD calculation and PCM(ACN) for ECD calculation) provided 2 most populated conformers (Figure S2).

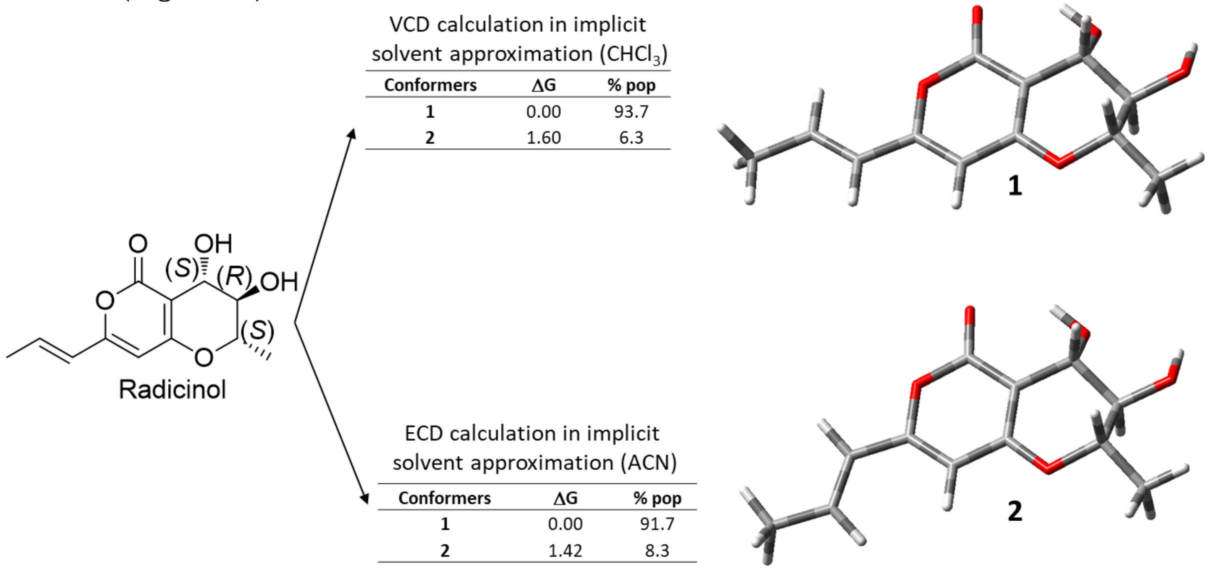

Figure S3. Calculated structures, relative ΔG energies (in kcal/mol units) and population percentages of radicinol (**3**). DFT/B3LYP/TZVP(PCM) level of theory.

VCD-IR experimental spectra were recorded in $CDCl_3$ at concentration of 0.2M. The experimental spectra were compared to the calculated ones at DFT/B3LYP/TZVP/PCM($CHCl_3$) level of theory (Figure S3).

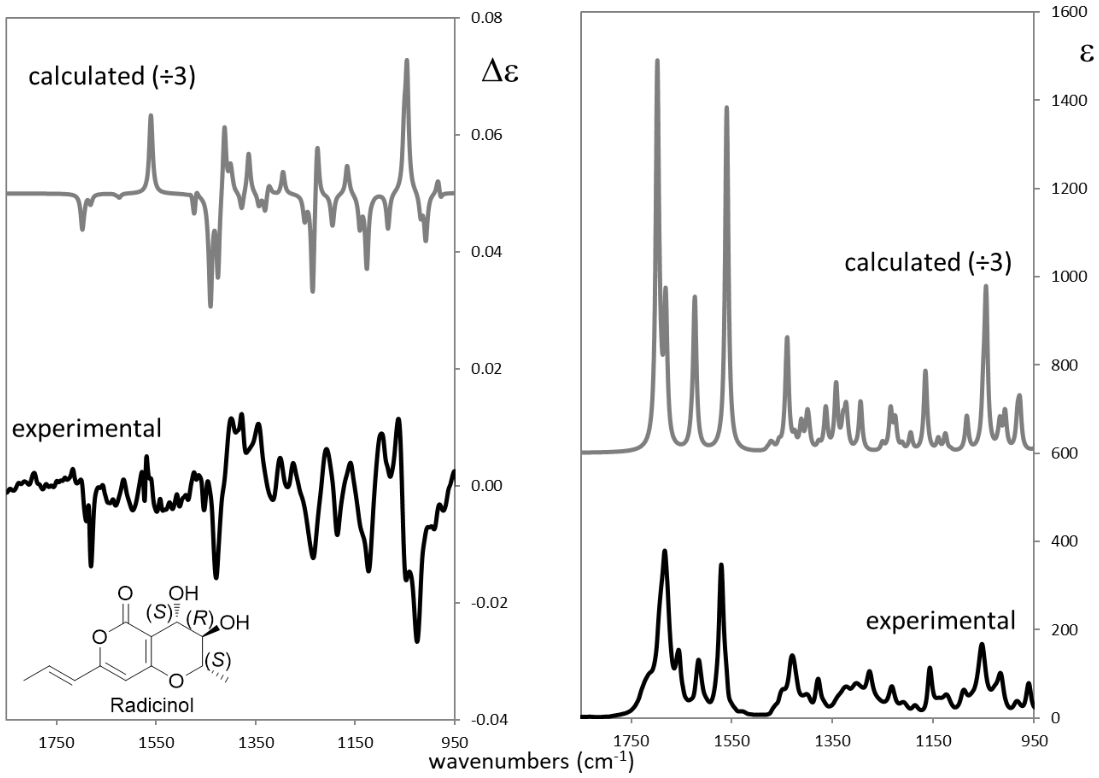

Figure S4. Comparison of experimental and calculated VCD (left) and IR (right) spectra of radicinol (**3**). DFT/B3LYP/TZVP/PCM($CHCl_3$) level of theory. 0.99 applied scaling factor.

ECD-UV experimental spectra were recorded in acetonitrile at concentration of 0.0051M. The experimental spectra were compared to the calculated ones at TD-DFT/CAM-B3LYP/aug-cc-pVDZ/PCM(ACN) level of theory (Figure S4).

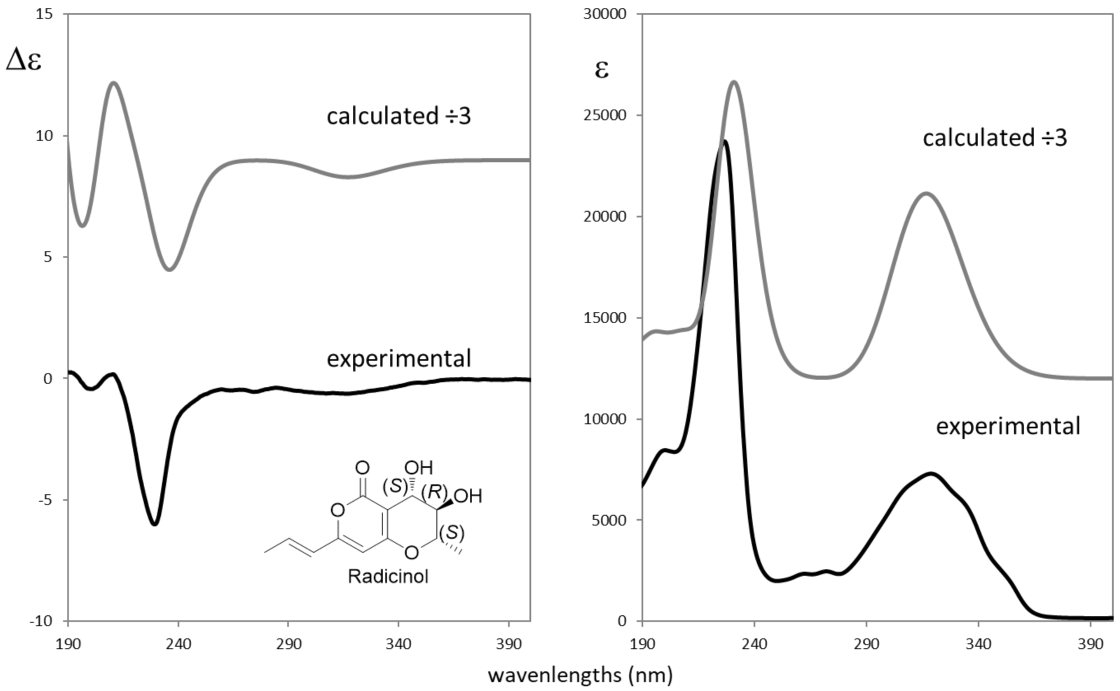

Figure S5. Comparison of experimental and calculated ECD (left) and UV (right) spectra of radicinol (**3**). TD-DFT/CAM-B3LYP/aug-cc-pVDZ/PCM(ACN) level of theory. Calculated spectra are 15 nm red-shifted.

ORD experimental spectrum was recorded in chloroform at concentration of 0.26 g/100mL. The experimental spectra were compared to the calculated ones at TD-DFT/B3LYP/aug-cc-pVDZ/PCM($CHCl_3$) level of theory (Figure S5).

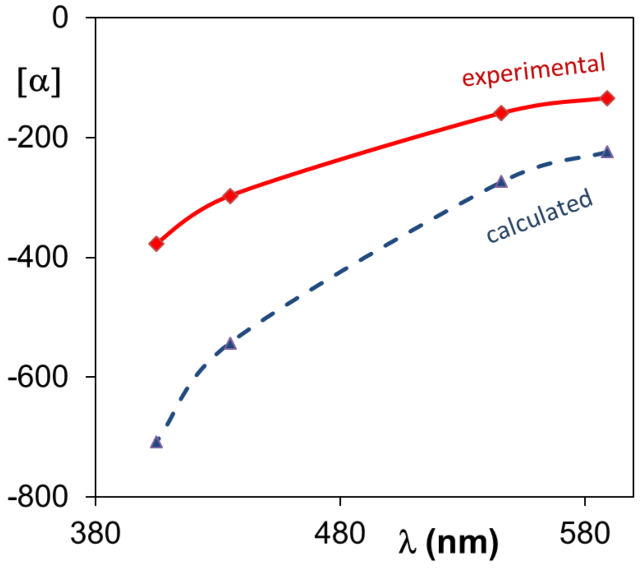

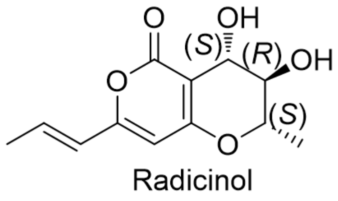

λ (nm)	Avg (ORD)	EXP. $CHCl_3$
**589**	-224	-133.9
**546**	-273	-159.2
**435**	-543	-297.3
**405**	-708	-377.2

Figure S6. Comparison of experimental and calculated ORD of radicinol (**3**). TD-DFT/B3LYP/aug-cc-pVDZ/PCM($CHCl_3$) level of theory.

Section SI-2. <u>AC of 3-*epi*-radicinin (**4**)</u>

Conformational search and DFT optimization at B3LYP/TZVP level of theory (PCM($CHCl_3$) was used for VCD and ORD calculation and PCM(ACN) for ECD calculation) provided 2 most populated conformers (Figure S6).

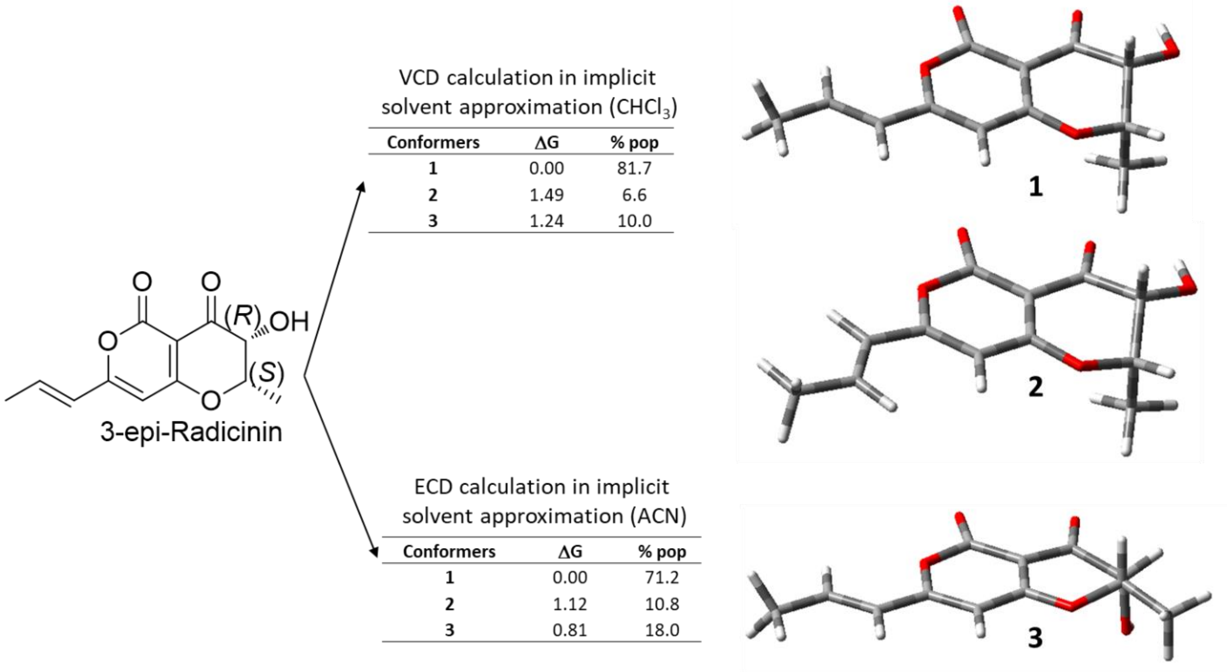

Figure S7. Calculated structures, relative ΔG energies (in kcal/mol units) and population percentages of 3-epi-radicinin (**4**). DFT/B3LYP/TZVP(PCM) level of theory.

VCD-IR experimental spectra were recorded in $CDCl_3$ at concentration of 0.37M. The experimental spectra were compared to the calculated ones at DFT/B3LYP/TZVP/PCM($CHCl_3$) level of theory (Figure S7).

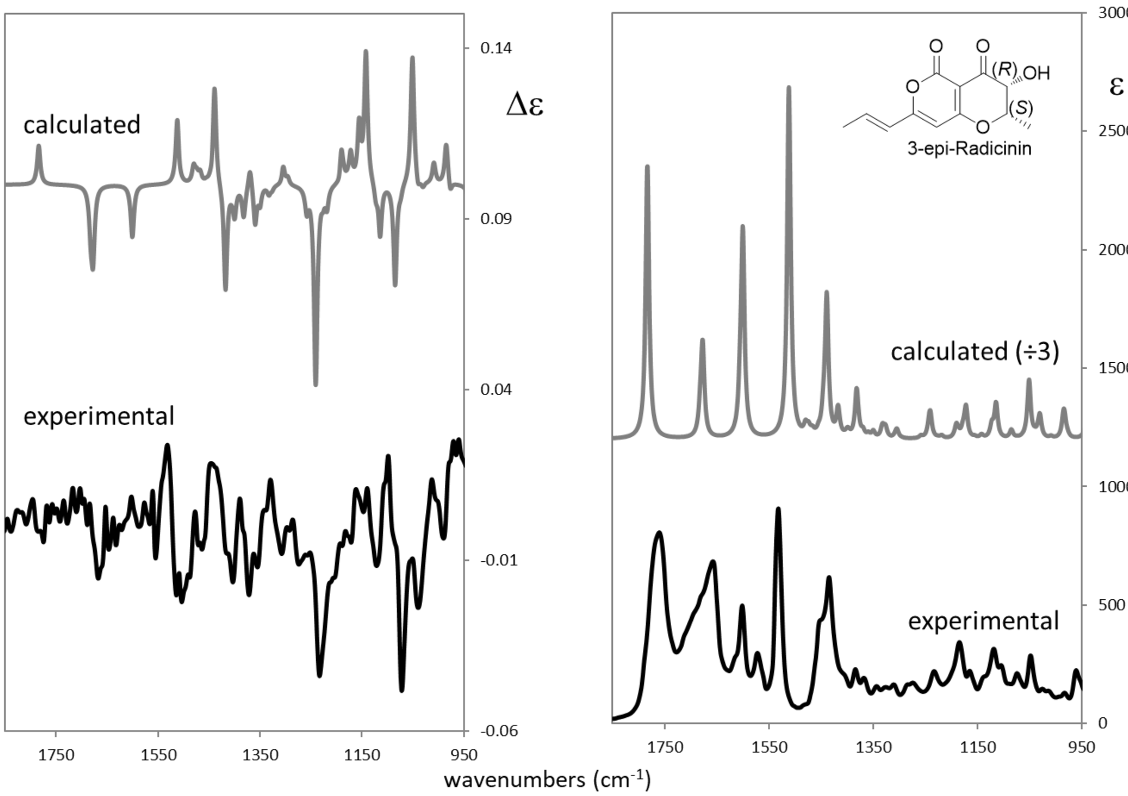

Figure S8. Comparison of experimental and calculated VCD (left) and IR (right) spectra of 3-*epi*-radicinin (**4**). DFT/B3LYP/TZVP/PCM($CHCl_3$) level of theory. 0.99 applied scaling factor.

ECD-UV experimental spectra were recorded in acetonitrile at concentration of 0.0053M. The experimental spectra were compared to the calculated ones at TD-DFT/CAM-B3LYP/aug-cc-pVDZ/PCM(ACN) level of theory (Figure S8).

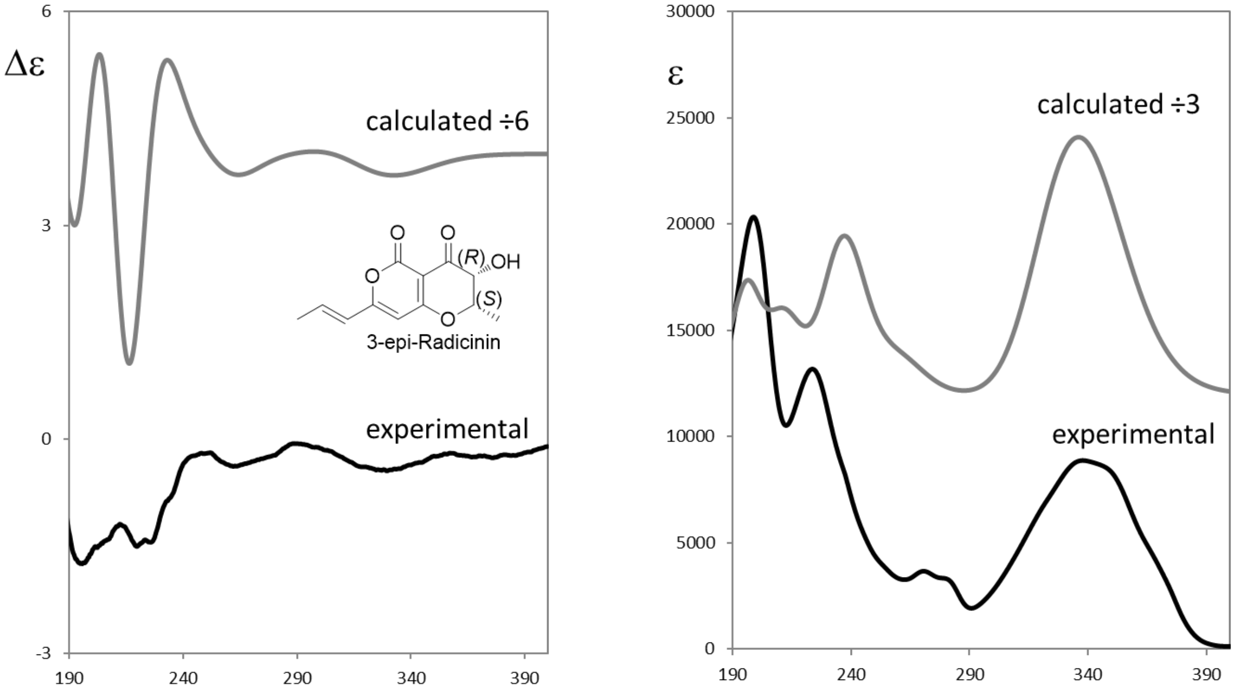

Figure S9. Comparison of experimental and calculated ECD (left) and UV (right) spectra of 3-*epi*-radicinin (**4**). TD-DFT/CAM-B3LYP/aug-cc-pVDZ/PCM(ACN) level of theory. Calculated spectra are 15 nm red-shifted.

ORD experimental spectrum was recorded in chloroform at concentration of 0.13 g/100mL. The experimental spectra were compared to the calculated ones at TD-DFT/B3LYP/aug-cc-pVDZ/PCM($CHCl_3$) level of theory (Figure S9).

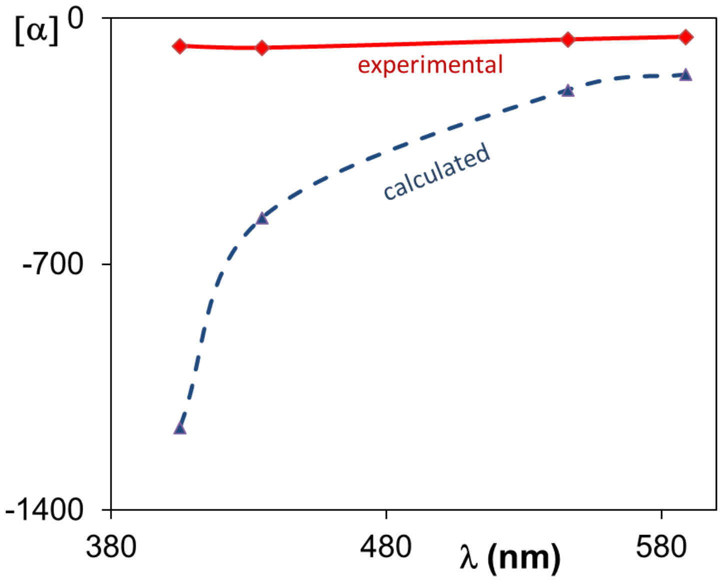

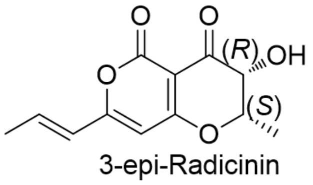

λ (nm)	Avg (ORD)	EXP. $CHCl_3$
**589**	-160	-52.52
**546**	-204	-60.2
**435**	-568	-83.712
**405**	-1167	-78.152

Figure S10. Comparison of experimental and calculated ORD of 3-*epi*-radicinin (**4**). TD-DFT/B3LYP/aug-cc-pVDZ/PCM($CHCl_3$) level of theory.

Section SI-3. <u>AC of 3-*epi*-radicinol (**5**)</u>

Conformational search and DFT optimization at B3LYP/TZVP level of theory (PCM($CHCl_3$) was used for VCD and ORD calculation and PCM(ACN) for ECD calculation) provided 2 most populated conformers (Figure S10).

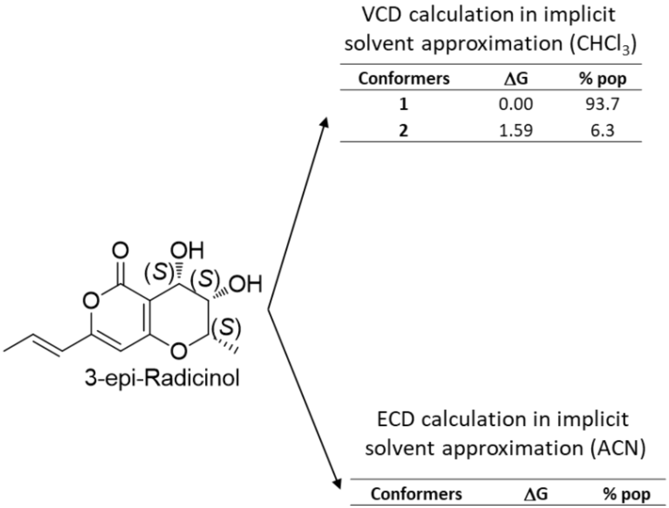

VCD calculation in implicit solvent approximation ($CHCl_3$)

Conformers	ΔG	% pop
**1**	0.00	93.7
**2**	1.59	6.3

ECD calculation in implicit solvent approximation (ACN)

Conformers	ΔG	% pop
**1**	0.00	92.0
**2**	1.45	8.0

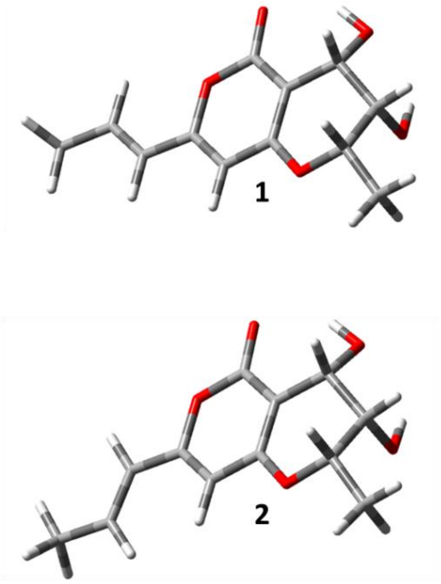

Figure S11. Calculated structures, relative ΔG energies (in kcal/mol units) and population percentages of 3-*epi*-radicinol (**5**). DFT/B3LYP/TZVP(PCM) level of theory.

VCD-IR experimental spectra were recorded in $CDCl_3$ and ACN-d_6 at concentration of 0.2M. The experimental spectra were compared to the calculated ones at DFT/B3LYP/TZVP/PCM($CHCl_3$) level of theory (Figure S11).

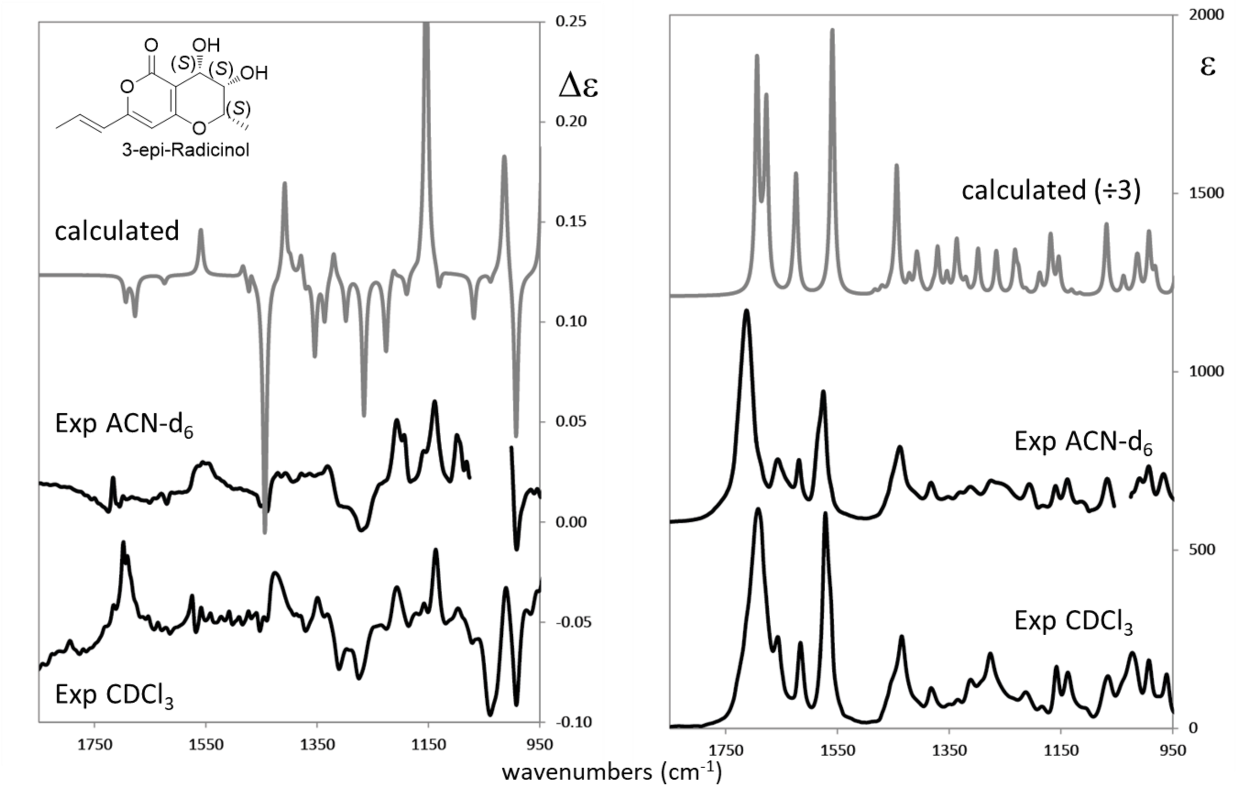

Figure S12. Comparison of experimental and calculated VCD (left) and IR (right) spectra of 3-*epi*-radicinol (**5**). DFT/B3LYP/TZVP/PCM($CHCl_3$) level of theory. 0.99 applied scaling factor.

ECD-UV experimental spectra were recorded in acetonitrile at concentration of 0.0051M. The experimental spectra were compared to the calculated ones at TD-DFT/CAM-B3LYP/aug-cc-pVDZ/PCM(ACN) level of theory (Figure S12).

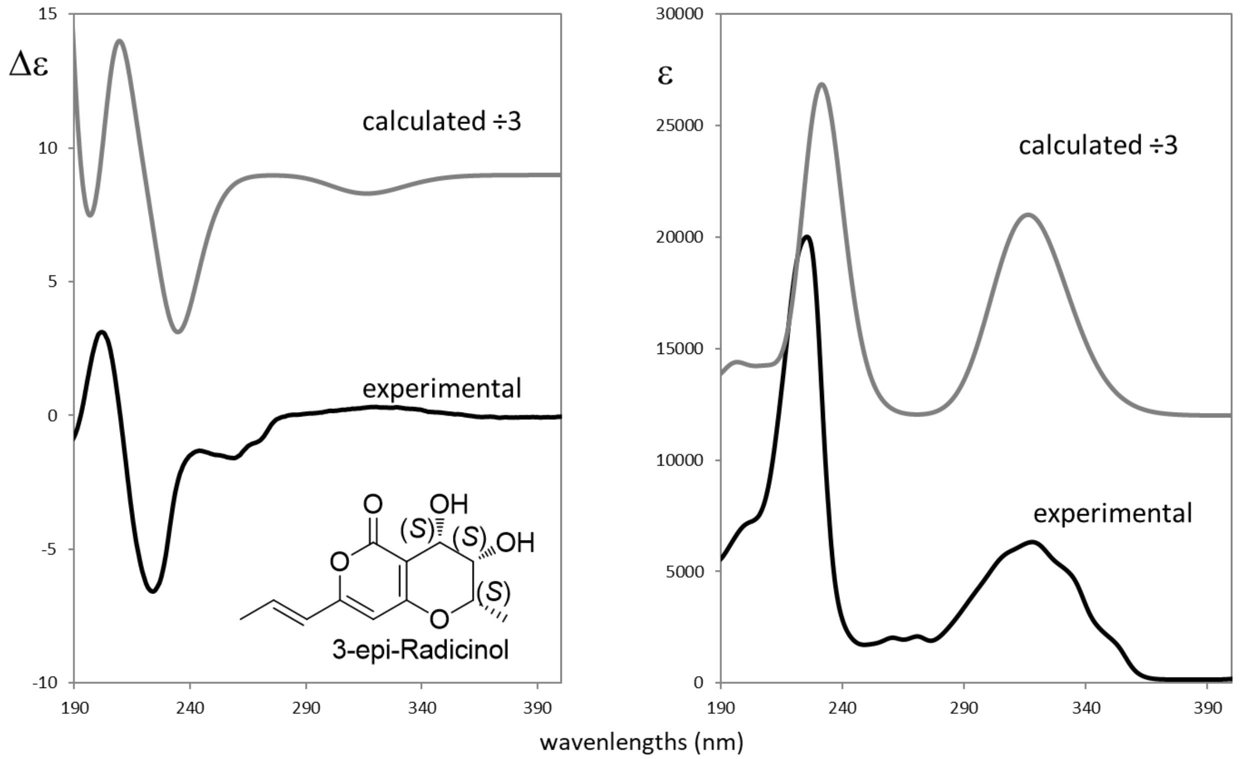

Figure S13. Comparison of experimental and calculated ECD (left) and UV (right) spectra of 3-*epi*-radicinol (**5**). TD-DFT/CAM-B3LYP/aug-cc-pVDZ/PCM(ACN) level of theory. Calculated spectra are 15 nm red-shifted.

ORD experimental spectrum was recorded in chloroform at concentration of 0.30 g/100mL. The experimental spectra were compared to the calculated ones at TD-DFT/B3LYP/aug-cc-pVDZ/PCM($CHCl_3$) level of theory (Figure S13).

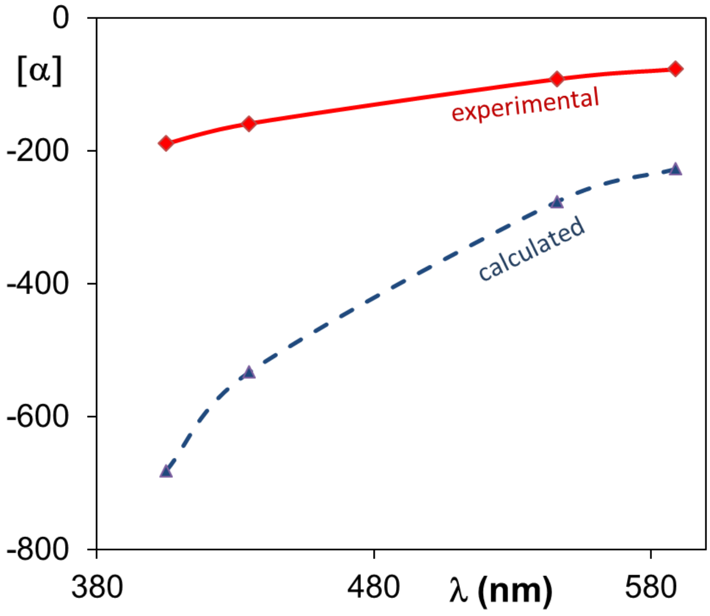

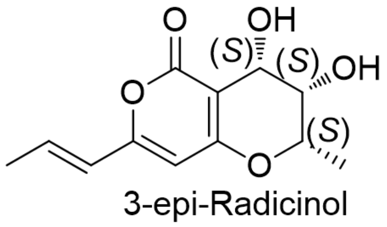

λ (nm)	Avg (ORD)	EXP. $CHCl_3$
**589**	-227	-76.6
**546**	-276	-91.7
**435**	-533	-158.8
**405**	-681	-189.3

Figure S14. Comparison of experimental and calculated ORD of 3-*epi*-radicinol (**5**). TD-DFT/B3LYP/aug-cc-pVDZ/PCM($CHCl_3$) level of theory.
